# Comparison of Genetic Structure of Epixylic Liverwort *Crossocalyx hellerianus* between Central European and Fennoscandian Populations

**DOI:** 10.1371/journal.pone.0133134

**Published:** 2015-07-17

**Authors:** Eva Holá, Jiří Košnar, Jan Kučera

**Affiliations:** Department of Botany, Faculty of Science, University of South Bohemia, České Budějovice, Czech Republic; Australian National University, AUSTRALIA

## Abstract

Patterns of genetic variation and spatial genetic structure (SGS) were investigated in *Crossocalyx hellerianus*, a strictly epixylic dioicous liverwort (Scapaniaceae *s*.*l*., Marchantiophyta). Studied populations were located in Fennoscandia and Central Europe, with localities differing in availability of substrate and the population connectivity, and their populations consequently different in size, density, and prevailing reproductive mode. A set of nine polymorphic microsatellites was successfully developed and used. Identical individuals were only found within populations. Especially in large populations, the majority of the individuals were genetically unique. Resampled number of genotypes, mean number of observed alleles per locus after rarefaction, and Nei’s gene diversity in large populations reached high values and ranged between 4.41–4.97, 3.13–4.45, and 0.94–0.99, respectively. On the contrary, the values in small populations were lower and ranged between 1.00–4.42, 1.00–2.73, and 0.00–0.95, respectively. As expected, large populations were found to be more genetically diverse than small populations but relatively big diversity of genotypes was also found in small populations. This indicated that even small populations are important sources of genetic variation in bryophytes and processes causing loss of genetic variation might be compensated by other sources of variability, of which somatic mutations might play an important role. The presence of SGS was discovered in all populations. Large populations possessed less SGS, with individuals showing a pronounced decrease in kinship over 50 cm of distance. Apparent SGS of small populations even at distances up to 16 meters suggests the aggregation of similar genotypes, caused predominantly by the deposition of asexually formed gemmae. Although no strong kinship was detectable at the distances over 16 meters in both small and large populations, identical genotypes were occasionally detected at longer distances (20–80 m), suggesting effective dispersal of asexual propagules.

## Introduction

The structure of genetic diversity on fine scales within populations and on larger scales among populations may bring valuable insights into the reproductive systems of studied organisms including the assessment of reproductive effort, rates of sexual and vegetative reproduction, dispersal capacity of diaspores and levels of gene flow among populations. Bryophytes are generally considered to possess high dispersal capacity of their sexually originating spores [1, 2], which however is often impaired by the relatively low reproductive effort allocated into the production of energetically costly sporophytes. In dioicous bryophytes, which constitute a significant proportion as opposed to the situation in remaining land plants [3, 4], the sexual reproduction is further complicated by the necessity of spatial proximity of male and female gametangia, as the dispersal range for sperm is generally very short [5, 6] On the other hand, most bryophytes also propagate by means of vegetative fragments, and a notable proportion of bryophytes produce specialized vegetative diaspores, such as the gametophytic gemmae, which were proven to possess a dispersal capacity comparable to spores and even effectively contributing to gene flow among populations [7]. Recruitment of progeny is nevertheless not only dependent on the formation and dispersal capacity of diaspores, but also on the diaspore establishment and sustainable growth conditions for mature plants. A significant proportion of bryophytes are known to be strictly specialized in particular substrates or habitats [8], and one such examples of habitat specialization are the epixylic species, i.e. species growing on decomposing wood matter. Decomposing wood supports a rich community of plants, fungi and animals [9]. Decaying logs are a very dynamic substratum with a non-random patchy distribution, restricted duration and time-variable quality [8, 10] where composition of bryophyte communities changes following the decay stage of logs [11]. Moreover, a sufficient amount of decomposing wood is missing from most human-managed forests and is only present in natural and old-growth forests. These unfortunately belong to prime examples of habitats under globally strong anthropogenic pressure [12]. Epixylic species are thus handicapped on two scales. The suitable substrate is not continuously available, as exemplified in a study of the epixylic liverwort *Ptilidium pulcherrimum*, which showed that less than 1% of produced spores were deposited on substrate suitable for establishment [13]. On the landscape scale, extensive forestry has resulted in considerable decrease and fragmentation of forest habitats, in which the specific substrate occurs. To date, no strictly epixylic bryophyte has been studied, although the genetic diversity and structure of epiphytic forest bryophytes has been addressed in several studies [14–16]. Genetic variation in wood living fungi and beetles, to our knowledge the only studied epixylic organisms, showed low gene flow and low genetic variation among isolated and fragmented populations similarly as it was the case in other forest dwelling species [16–18].

Recent studies of population genetic variability and spatial genetic structure using DNA fingerprinting methods have shown a remarkable variability of results, showing the uniqueness of parameters of individual reproduction systems in different taxa. One of the most interesting findings is that the level of genetic differentiation among bryophytes reproducing mostly or exclusively vegetatively was in several cases surprisingly high [19–21]. The genetic variability in mostly non-sexual populations can be maintained by migration from neighboring populations, occasional sporophyte production, or by the accumulation of somatic mutations [19, 20, 22]. Studies of spatial genetic structure (SGS) in bryophyte populations are also relatively rare [14, 23–26]. Only one study [23] focused on small-scale pattern of SGS in the liverwort species *Barbilophozia attenuata* Mart. (Loeske), which is a species closely related to our object of study, possessing a similar reproduction mode.

**Fig 1:**
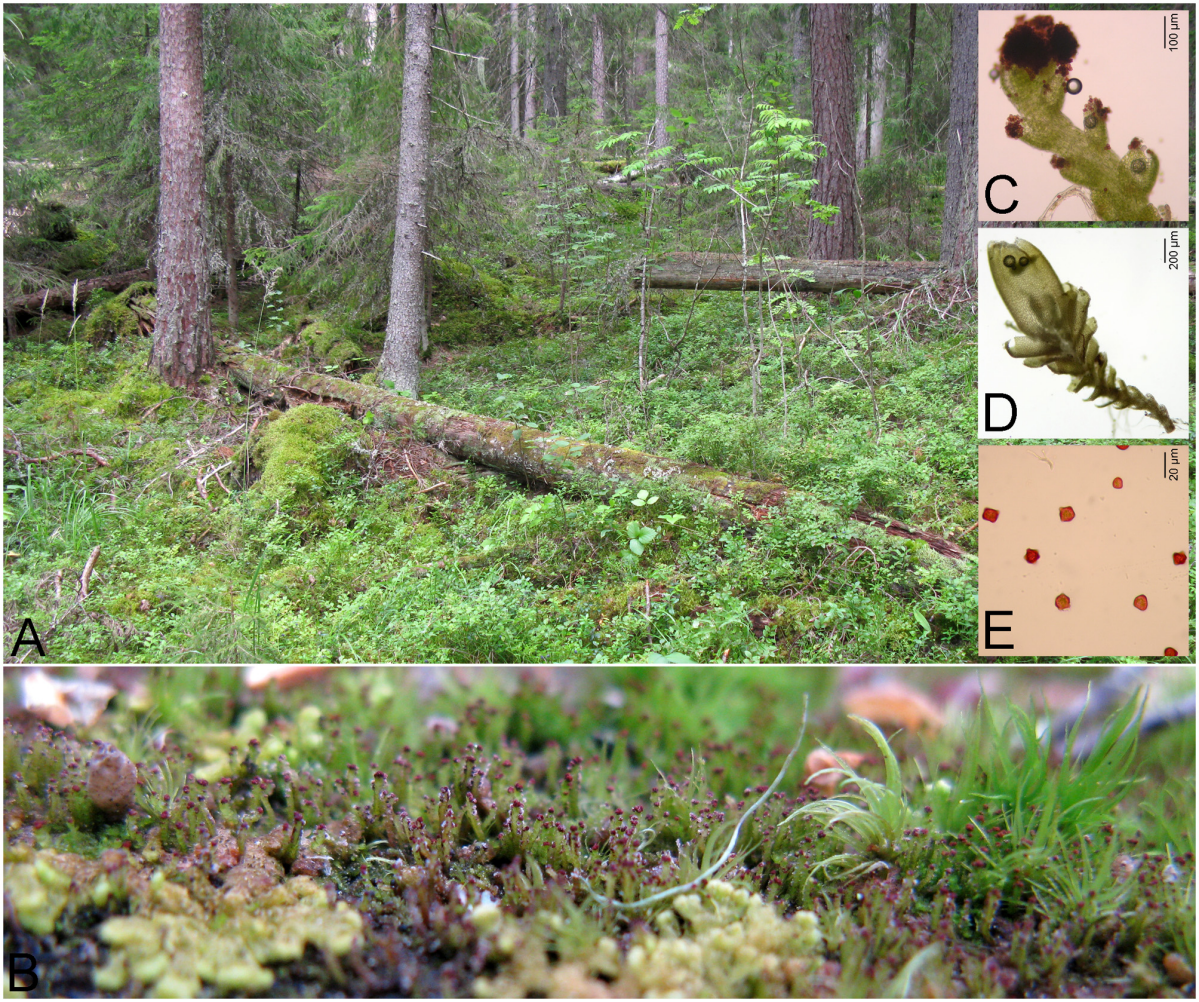
The studied species *Crossocalyx hellerianus*. Pictures from Vesijako Strict Nature Reserve (A) overgrown log of *C*. *hellerianus*, (B) *C*. *hellerianus* in detail. Light microscope pictures (C) gemmiparous shoot, (D) perianth, (E) gemmae.

We have studied *Crossocalyx hellerianus* (Nees ex Lindenb.) Meyl., a minute, circumboreally distributed dioicous epixylic liverwort (Fig 1) of the family Scapaniaceae *s*.*l*. (Anastrophyllaceae [27, 28]). Both sexually formed spores and asexual gemmae are produced, with both being approximately 10–12 μm in diameter. Sexual reproduction is described as occasional in Nordic countries (sporophyte formation was observed in 2.5–12% of the colonies [29]), whereas in other parts of European distribution area it might be much rarer, e.g. they were never reported from Ireland and Britain [30, 31]. On the contrary, gemmae are always present and generally abundant. It is considered to be a colonist species with the potential life span of only a few years [32], inhabiting decaying logs (mostly of spruce) of intermediate decay stages [33]. With respect to its habitat preference, it usually occurs in old-growth spruce forests with high amounts of coarse woody debris [33] and therefore it is relatively rare in all parts of its distribution area. In the countries of this study, it has been classified as Near Threatened (NT) in Finland [34], and Endangered (EN) in the Czech Republic [35] according to IUCN criteria. In the latter country, only 8 populations are recently known, with only one population classified as large (see below for definitions).

The study populations, located in Scandinavia and Central Europe, differ in size, density, prevailing reproductive mode, and population connectivity. Thus, these populations represent a suitable study system for investigations on patterns of genetic variation with regard to the above mentioned population characteristics. The studied liverwort moreover produces sexual and vegetative diaspores of potentially very similar dispersal capacities with respect to their size, which facilitates the inference on dispersal efficiency. We hypothesized that the population size or density and prevailing reproductive mode would be mirrored in the population genetic diversity and fine-scale spatial genetic structure. Microsatellite markers, which have been developed for his study, further allowed for the assessment of gene flow levels among populations and rates between sexual and asexual reproduction.

## Material and Methods

### Study sites and sampling

Sampling was performed in Finland (FI, 4 populations) and in the Czech Republic (CZ, 6 populations; Table 1 and S1 Fig). Mean geographic distances among CZ populations amounted to 55 km, those among FI populations 62 km, and the distances among CZ and FI populations averaged 1500 km (S1 Fig). Studied Finnish populations are located in the boreal zone of southern Finland, representing only a part of regional populations [34]. Czech populations are located in South Bohemia within the temperate zone and represent all known Czech localities as of 2012. The Finnish forests are mainly old virgin forests dominated by spruce with several canopy layers (pines, birches and aspens), characterized by huge amounts of decaying conifer wood, which is reflected in the relatively common occurrence of *Crossocalyx hellerianus*. The Czech forests represent small extant fragments of herb-rich and acidophilous montane mixed old-growth forests with the tree composition and herb vegetation approaching the natural one, dominated mostly by beech with spruce admixtures. The amount of suitable decaying wood is only high in the Boubínský prales National Nature Reserve among the Czech forests. Consequently, *C*. *hellerianus* is relatively common only in this reserve, while the other Czech localities support only very small populations of the liverwort (Table 1).

**Table 1:** List of study populations with quantitative data.

Locality abbr.	Population	Coordinates [WGS 84]	Country	Population size	Number of sampled logs	Date of sampling (DD.MM.YY)
Z	Boubínský prales National Nature Reserve	48°58'32"N, 13°48'54"E	CZ	LARGE	9	17.11.12
G	Kamenná hill	48°49'08"N, 13°48'50"E	CZ	SMALL	3	22.11.12
M	Medvědí hora Nature Monument	48°37'13"N, 14°13'40"E	CZ	SMALL	2	16.09.12
Y	Milešický prales Nature Reserve	48°59'06"N, 13°50'19"E	CZ	SMALL	5	17.11.12
R	Nová Bystřice	49°01'13"N, 15°01'16"E	CZ	SMALL	1	08.05.12
P	Žofínský prales National Nature Reserve	48°40'10"N, 14°42'20"E	CZ	SMALL	2	13.10.12
N	Nuuksio National Park	60°18'36"N, 24°29'57"E	FI	LARGE	8	11.08.12
S	Sudenpesänkangas Nature Reserve	61°12'15"N, 25°11'49"E	FI	LARGE	8	08.08.12
K	Kotinen Nature Reserve	61°14'28"N, 25°03'47"E	FI	LARGE	8	08.08.12
V	Vesijako Strict Nature Reserve	61°21'00"N, 25°06'04"E	FI	LARGE	8	09.08.12

In populations, where *C*. *hellerianus* was abundant (with more than 10 logs supporting the species, further on assigned as ‘large’ populations, Table 1), 8–9 logs were sampled. In smaller populations (‘small’, Table 1), all logs supporting the occurrence of *C*. *hellerianus* were sampled and surroundings of these logs (up to 0.5 km around) were investigated for possible occurrence.

**Fig 2:**
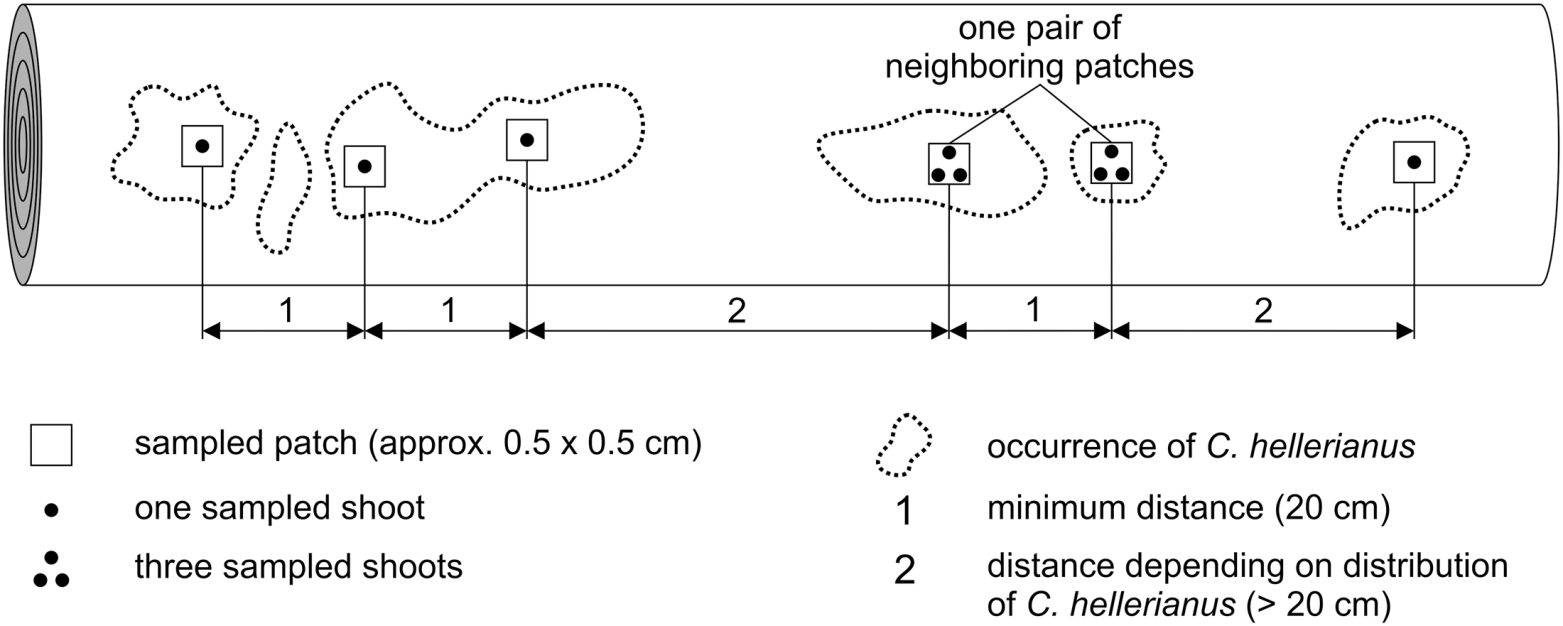
Schematic illustration of *Crossocalyx hellerianus* sampling on logs.

Approximately 0.5×0.5 cm was sampled from every occurrence of *C*. *hellerianus* at a minimum distance of 20 cm; the maximum distance depended on the patchy distribution of species on each sampled log (Fig 2). For detection of genetic structure at the smallest spatial distances, three shoots were taken from four pairs of neighboring patches (one pair on each log) in large populations and three shoots from two pairs of neighboring patches in small populations. One shoot was taken from each of the other patches. Distances among shoots that originated from the same patch were arbitrary equaled to one centimeter and distances among the sampled patches were measured. The small size of the population Nová Bystřice (10×15 cm) allowed for removal of only five shoots.

All studied populations were searched for the production of sporophytes. As these are ephemeral and we were not able to record them at the time of visit, perianths were considered as the indication of the sexual reproduction. Perianths (Fig 1D) of the leafy liverworts are gametophytic structures of foliar origin around the archegonium which serve the protection of developing capsule. Perianths were searched in all sampled patches, using a stereo-microscope.

### Ethics statement

All necessary permits were obtained for field studies to collect species material. Metsähallitus issued the permission for entry into Finish localities and Nature Conservation Agency of the Czech Republic issued the entry permission into Czech localities. No special permission is required for sampling of *Crossocalyx hellerianus* in the respective countries, although it is considered is Endangered (EN) species in the Czech Republic according to IUCN criteria [32], which however does constitute the basis for legal protection in that country (see S1 File). The species sampled are not listed by CITES (Convention on the International Trade in Endangered Species). All studied localities are on public lands.

### Genetic analysis

A SSR-enriched genomic library was constructed using a biotin-streptavidin capture method [36]. Screening of SSR-enriched genomic library was performed using combined approach involving traditional cloning and Sanger sequencing of the library, together with direct 454 pyrosequencing of the library on a GS Junior System (454 Life Sciences, Branford, USA) as described in [37]. Specific primers were designed using Primer3 [38, 39], see Table 2.

**Table 2:** Characterization of the nine microsatellite loci developed for *Crossocalyx hellerianus*.

Repeat motif	Ta [°C]	Forward primer (5'- 3')	Reverse primer (5'- 3')	No. of alleles	Size range [bp]	GenBank accession no.
(TG)13	58	CCACTTTCCATTGTGACCTTT	AGTTTCTTCTCCGCCATCA	7	148–160	KM065844
(AC)10	54	GGACGCACTAACTCGTTTTCTC	GGTCCAGCATGAGGTTGATT	33	246–314	KM065843
(TG)24	54	TTCTGTCATTTTCGGATTTGG	GTGGGCAACTTCTTTGGACT	18	384–426	KM065842
(TC)24	54	TTGGGATGAGAAAAGTGA	CCTCGTATTGATTGTGGGTAT	24	486–536	KM065838
(GT)10	54	CCTTGCAGCTCATATCTTGTT	CCTTTCGTCCACCATAAGTCC	14	205–237	KM065837
(CA)11	54	CCAAGCATGAACTAATCCCATC	GCAAAGGTAACACCAAAGTGAG	5	158–172	KM065839
(CA)21	58	TCAAGAACCTTACATCCAAACC	GCATCACTCACTCCTCACCA	25	307–357	KM065840
(AC)13	54	CGTGGAAAGACTGTTGAGGA	GGATTTGAGGCGAGGGATAG	7	173–185	KM065845
(GT)13	54	CAAGCCAACAAGGAGAGAGATT	AAGCCCAATGTGAAGAAGGA	12	226–260	KM065841

Total genomic DNA was extracted from each of the analyzed shoots using the NaOH method [40]. PCRs were performed in a reaction mixture containing 0.5 μL of genomic DNA, 2.5 mM MgCl_2_, 0.2 mM dNTPs, 0.3 μM primers, 0.25 U *Taq* polymerase (Top-Bio, Prague, Czech Republic) in the manufacturer’s reaction buffer, and sterile water to make up a final volume of 5 μL. Amplifications were performed with an initial denaturation of 3 min at 94°C, followed by 45 cycles of 1 min denaturation at 94°C, 30 s at primer-specific annealing temperature (Table 2), 15–30 s extension at 72°C, and a final extension of 10 min at 72°C. PCR products were pooled and analyzed using fragment analysis on an ABI 3730xl DNA Analyser (Applied Biosystems) with GeneScan 600 LIZ (Applied Biosystems, Foster City, USA) as the internal size standard. Microsatellite alleles were scored using GeneMarker v1.80 (SoftGenetics LLC, State College, USA) and were coded as a number of repeats of the SSR motif. Samples in which amplification of more than three loci failed were omitted. Allelic data are available in S2 File.

### Data analysis

Nei’s gene diversity (Ĥ) was calculated using Arlequin v3.5 [41]. Number of genotypes—N_g_ and number of recurrent genotypes—N_rg_ were calculated in the GenClone 2.0 program [42]. With respect to different sample size of populations, values of N_g_ were resampled using GenClone 2.0, and HP-Rare software [43] was used for rarefaction of mean number of observed alleles per locus—N_a_. For both N_g_ and N_a_ calculations, the sample sizes were adjusted to five (the smallest sample in the comparison). The probability that individuals shared the same multilocus genotypes (MLG) were derived from sexual reproduction involving recombination (P_sex_) calculated in the GenClone 2.0. Samples with missing data were excluded from all above mentioned computations.

In addition to P_sex_ assessment, linkage disequilibrium analysis was performed to assess whether marker distributions resulted from sexual or asexual reproduction. Multilocus linkage disequilibrium was tested using the index of association modified to remove the effect of number of loci analyzed (r_d_ [44]) and calculated for each population using Multilocus v 1.3. Significance was tested by comparing the observed dataset against the null hypothesis of infinite amount of sex and recombination by random shuffling the alleles amongst individuals using 1,000 randomizations.

Hierarchical structure of genetic variation was examined using analysis of molecular variance (AMOVA) in Arlequin v 3.5 [41] with calculations based on the R_ST_-like method, using the sum of squared size differences. The R_ST_-like method was preferred because a preliminary allele permutation test performed in SPAGeDi 1.4 software [45] was significant, indicating that an allele size-based statistic was informative for population differentiation and may contain more information than allele identity measures such as F_ST_, which is likely to provide a biased estimate of gene flow [46]. The following partitioning of genetic variation was tested: between distant geographic regions (Czech Republic and Finland) and among localities within the regions. The analysis based on F_ST_-like method showed that variation among populations within regions was slightly higher than variation between the two geographic regions (CZ vs. FI). In addition, the pairwise R_ST_ values for all populations were computed. The significance of AMOVA components and of pairwise R_ST_ values was tested using 10,000 permutations.

To reveal the fine-scale spatial genetic structure (SGS), a spatial autocorrelation analysis was conducted in SPAGeDi 1.4 software [45]. Distance classes with upper boundaries of 0.01, 0.5, 1, 2, 4, 8, 16 and 500 m were used (spatial sampling information is available in S3 File). Multilocus pairwise kinship coefficients (F_ij_) based on Nason’s kinship coefficient [47] were calculated. To test the influence of population size on SGS, populations were further assigned into three groups: small CZ populations, large FI populations and the large CZ population (see Table 1). For each group of populations, mean multilocus pairwise kinship coefficient values were plotted against the upper boundaries of geographic distance classes. Significance of the mean F_ij_ per distance class was tested using 1,000 random permutations of individuals.

The spatial extent of clonal dispersal was quantified using distance classes and population assignment defined as above. The percentage of clones within each of the distance classes was calculated using pairwise comparisons which included identical genotypes and they were plotted against the upper boundaries of classes. In addition, the maximum distance among samples of the same genotype was recorded for each population.

## Results

### Population genetic analyses

**Fig 3:**
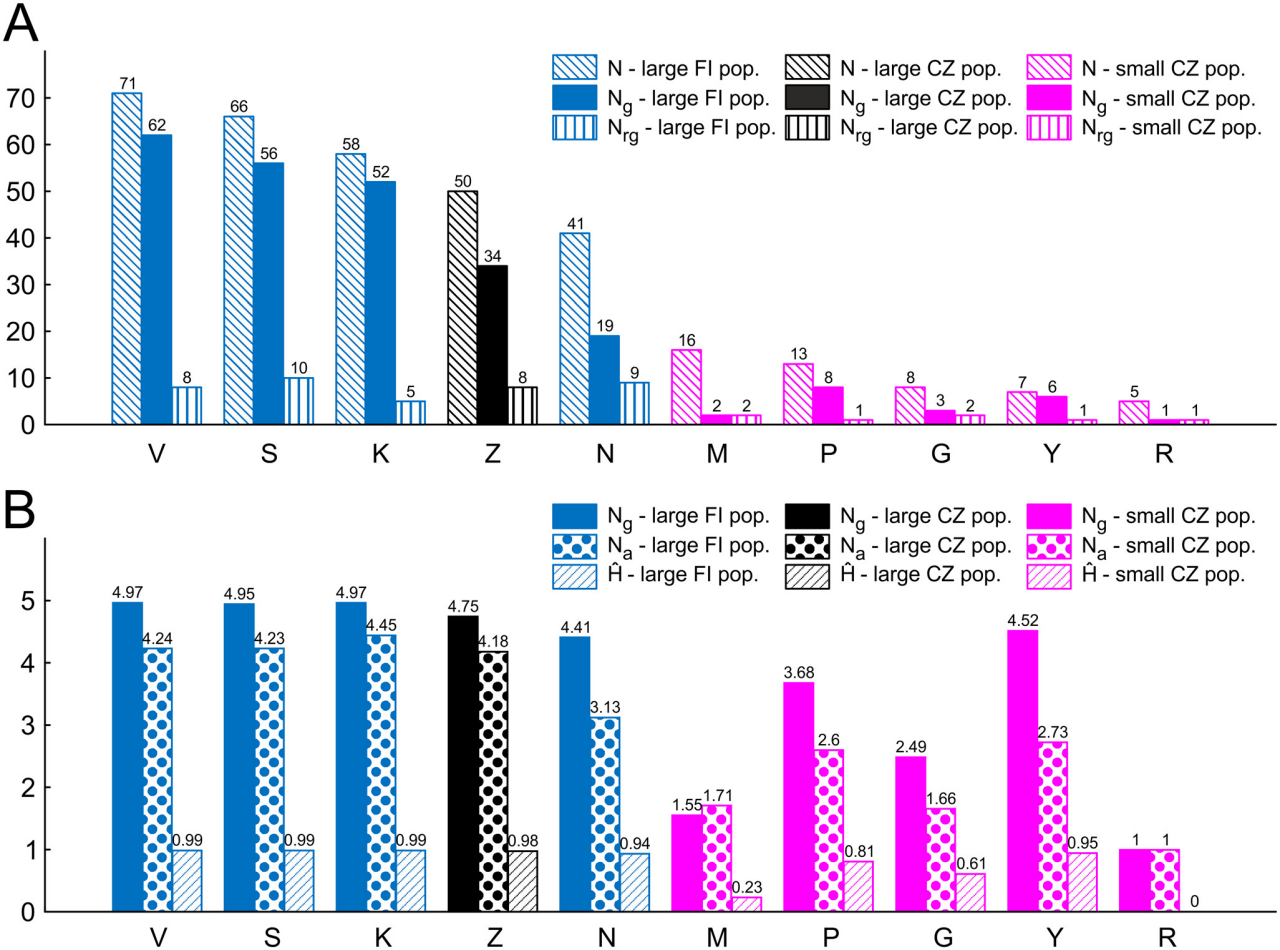
Genetic diversity indices for *Crossocalyx hellerianus* populations. (A) Sample size (N), number of genotypes (N_g_) and number of recurrent genotypes (N_rg,_ i.e. those occurring more than once) computed for all samples in each population. (B) Resampled values of number of genotypes (N_g_), mean number of observed alleles per locus (N_a_) after rarefaction, and Nei’s gene diversity (Ĥ). Abbreviations of localities correspond to Table 1.

Nine polymorphic microsatellite markers from the liverwort *Crossocalyx hellerianus* were developed (Table 2 and S2 Fig). All genotyped material was haploid and the microsatellite loci contained between 5 and 33 alleles (Table 2). The final dataset of 393 successfully genotyped samples contained two samples with missing data for three loci, four samples with missing data for two loci, and 52 samples with missing data for one locus, respectively. 243 MLGs were found among the 335 genotyped individuals (without missing data). Identical genotypes were only rarely detected inside large FI and CZ populations, while in small CZ populations recurrent genotypes occurred at higher rates (Fig 3 and S3 Fig). Identical genotypes were relatively frequently detected only within individual logs (see below). No identical genotype has been found among populations.

Resampled number of genotypes (N_g_), mean number of observed alleles per locus (N_a_) after rarefaction, and Nei’s gene diversity (Ĥ) varied from 1.00 to 4.97, 1.00 to 4.45, and 0.233 to 0.995, respectively (Fig 3). Lower values of N_a,_ N_g_ and Ĥ were detected in small CZ populations; the small CZ population R contained a single MLG.

The analysis of molecular variance based on R_ST_-like method (Table 3) showed that the highest proportion of genetic variation occurred within populations (67.7%), followed by the variation between the two geographic regions (CZ vs. FI; 25.3%), and the variation among populations (7.0%). Separate analyses of both regional datasets found higher rate of variation among CZ populations (18.5%) than among FI populations (6.2%).

**Table 3:** The distribution of genetic variation based on the analysis of molecular variance (AMOVA).

Source of variation	d.f.	Variance component	Variance %	Fixation index
Between CZ and FI groups of populations	1	53.5	25.3	F_CT_ = 0.253**
Among populations within groups	8	14.9	7	F_SC_ = 0.094***
Within populations	382	143.3	67.7	F_ST_ = 0.323***
Total (CZ and FI)	391	211.7		
Among CZ populations	5	21.9	18.5	F_ST_ = 0.185***
Within CZ populations	129	96.8	81.5	
Total (CZ)	134	118.7		
Among FI populations	3	11.8	6.2	F_ST_ = 0.062***
Within FI populations	254	180.0	93.9	
Total (FI)	257	191.8		

Significance of *F* values is marked as *** *P* < 0.001; ** *P* < 0.01.

**Fig 4:**
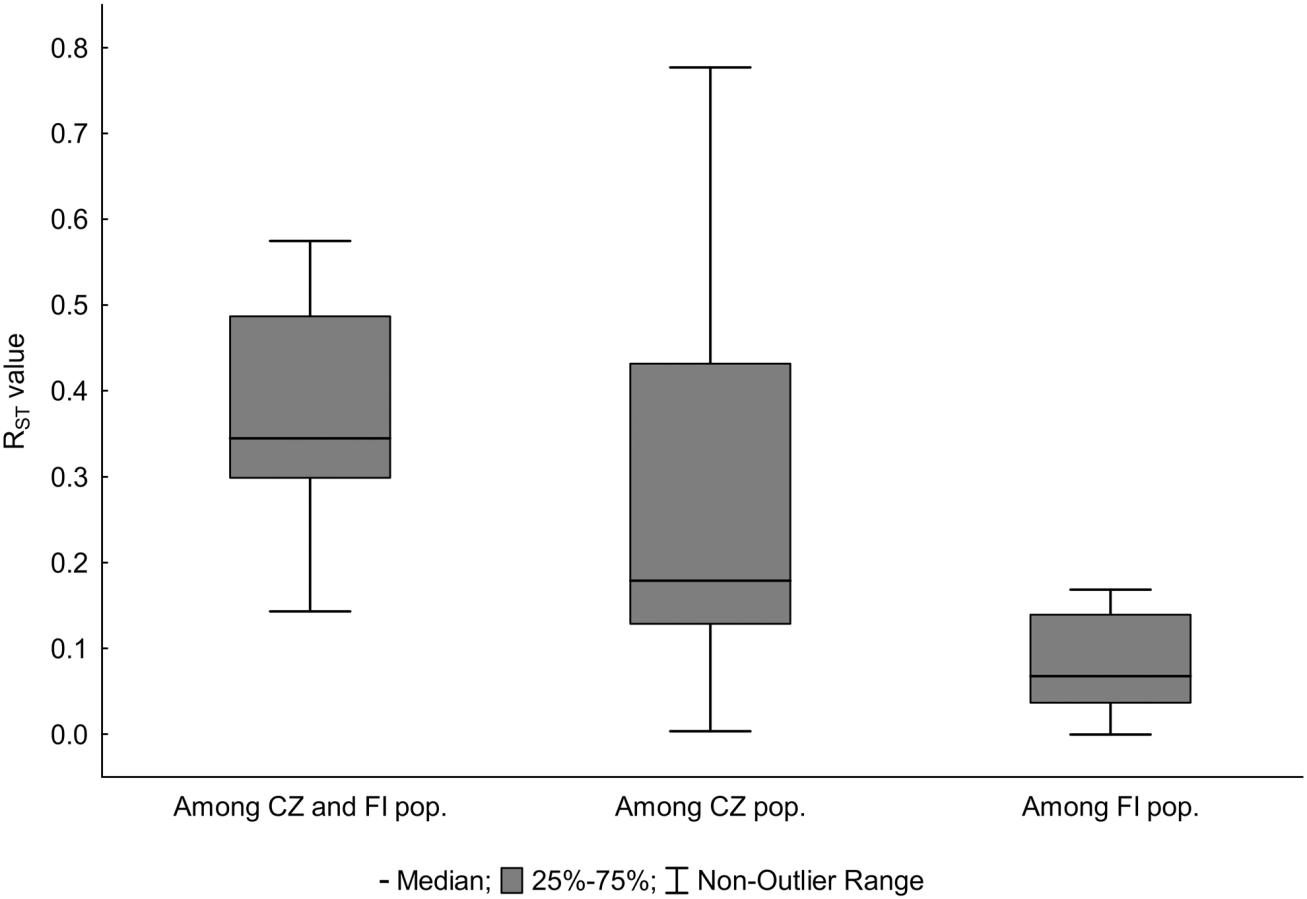
Genetic differentiation. Genetic differentiation among populations between and within the two geographic areas based on pairwise R_ST_ values.

The highest pairwise R_ST_ values were usually observed between CZ and FI populations (Fig 4 and S1 Table), which is in agreement with geographic distances separating both regions (ca. 1,500 km). Nevertheless, considerable divergence was also found among most of the CZ populations, with pairwise R_ST_ values usually higher than 0.1 (11 out of 15 values). On the contrary, the pairwise R_ST_ values between FI populations except the most remote population N did not exceed the value of 0.1. Even in case of population N, the pairwise comparisons with the remaining FI populations (K, S and V) revealed generally lower R_ST_ values than those observed among CZ populations separated by even shorter geographic distances (the distances between N and other FI populations spanned 106–120 km, whereas 18–92 km separated CZ populations, respectively). The pairwise R_ST_ values among geographically close populations (separated by distances not exceeding 18 km, i.e. CZ populations G, Z, Y, and FI populations S, K, V, respectively) were higher among CZ populations (see S1 Fig).

Significant and high r_d_ values indicating linkage disequilibrium were found in all small CZ populations (Table 4). Only the value for the large FI population N was comparable to values of small CZ populations. Non-significant or low values of linkage disequilibrium were observed in populations with high N_g_, N_a_, Ĥ. These populations also contained a high number of observed patches with perianths, indicating the production of sporophytes.

**Table 4:** Linkage disequilibrium, maximum distance between the same MLG, % of patches with perianths.

Locality	Linkage disequilibrium (r_d_)	Max. distance between samples of the same genotype [m]	% of patches with perianths
Z	0.06***	6.8	7.5
G	0.44***	15	0
M	0.87***	10	8.3
Y	0.20***	0.01	0
R	–	0.01	0
P	0.26***	3.5	0
N	0.22***	50	25.9
S	0.01	20	8.6
K	0.03**	62	24.5
V	0.02	80	25

Linkage disequilibrium (significance of r_d_ values is marked as *** *P* < 0.001; ** *P* < 0.01) based on data at nine microsatellite loci in *Crossocalyx hellerianus*, maximum distance between samples of the same multilocus genotype, and percentage of patches with perianths. Locality R comprised a single multilocus genotype.

### Spatial genetic structure

**Fig 5:**
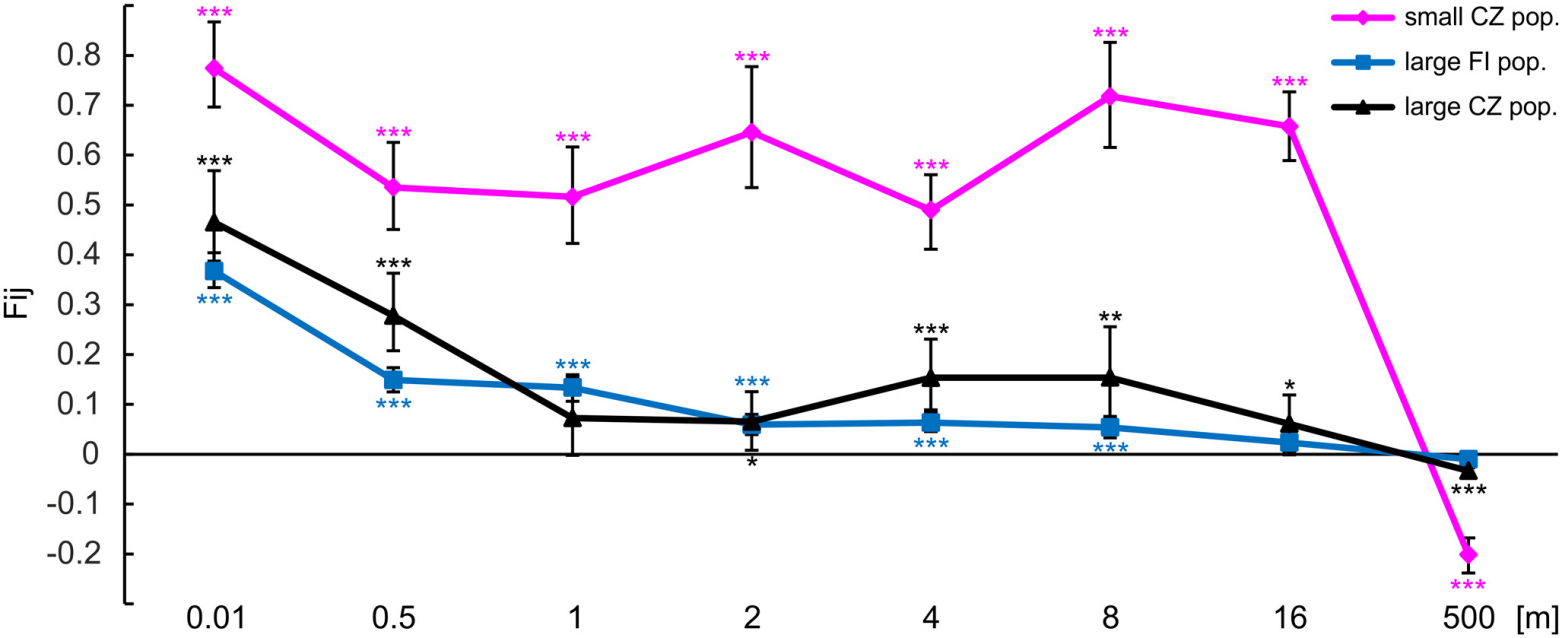
Spatial autocorrelation analysis based on microsatellite data. Populations of *Crossocalyx hellerianus* were divided into three categories (Table 1): small CZ pop., large FI pop., large CZ pop. The Nason’s kinship coefficients (F_ij_) are positioned along the X-axis at the mean pairwise distance within each distance class. Vertical bars show standard errors. Significance of average F values is marked as *** *P* < 0.001; ** *P* < 0.01; * *P* < 0.05.

Kinship coefficient in small CZ populations reached initial values of 0.77 on distances up to 1 cm, and varied from 0.48 to 0.72 on distances between 50 cm and 16 m (Fig 5). On the other hand, kinship coefficients in large populations were considerably lower, reaching the initial values of 0.47 and 0.37 on distances up to 1 cm, respectively, and varied from 0.02 to 0.28 on distances between 50 cm and 16 m. On distances exceeding 16 m, the kinship coefficient decreased and dropped below zero in all population groups.

**Fig 6:**
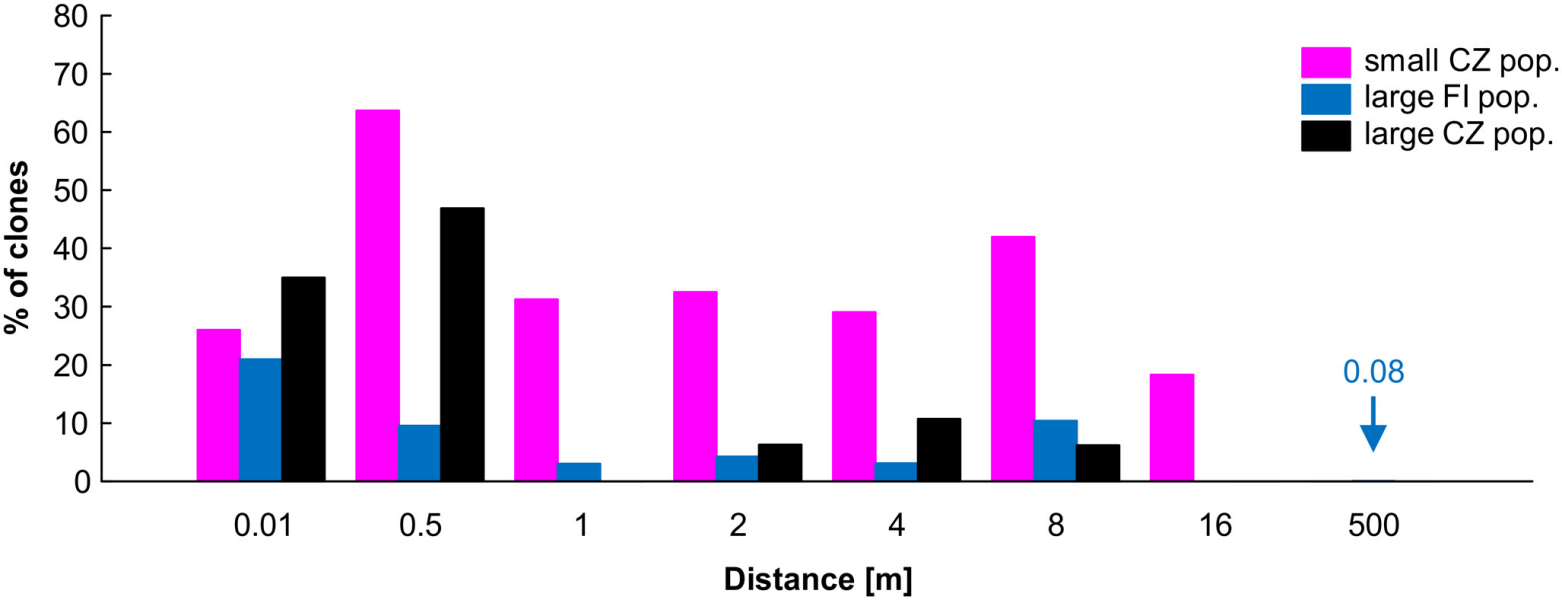
Percentages of clones within distance classes. Number of all pairwise comparisons in each distance: 0.01 m– 58, 96, 24; 0.5 m– 54, 211, 35; 1 m– 43, 166, 30; 2 m– 31, 274, 39; 4 m– 75, 321, 39; 8 m– 36, 266, 48; 16 m– 87, 168, 48; 500 m– 270, 6558, 948 for small CZ pop., large FI pop., large CZ pop., respectively. Long distances among clones (> 16 m) were found only in all FI populations (blue arrow).

The spatial extent of clonal dispersal differed between small and large populations (Fig 6 and S4 Fig). In small CZ populations, the percentage of pairwise comparisons with observed identical genotypes sustained high values (31.0–75.9%) for the first six distance classes (1 cm– 8 m), and started to decrease at the distances exceeding 16 m. The pattern found in large FI and CZ populations were rather similar to each other. High initial values of clonality were observed only in the first two distance classes (< 1 and 1–50 cm), then suddenly dropped in the third class (50–100 cm), and decreased more or less gradually at longer distances. However, the percentage of clonality was higher in the large CZ population than in all large FI populations. The probability of sexual origin (P_sex_) was relatively high for some of the putative clones from small CZ populations, but negligible for majority of individuals from large CZ and FI populations (S4 Fig).

The maximum extent of clonal dispersal was found in the population V, with shoots sharing the same genotype separated by 80 m (Table 4). Nevertheless, considerably long distances among shoots of identical genotypes (≥ 20 m) were found in all FI populations sampled (see Table 4). The maximum distance value for small CZ population was 15 m in population G, with clones always confined to one log.

## Discussion

### Genetic variability

The observed pattern of genetic variation in studied populations of *Crossocalyx hellerianus*, as documented by values of N_g_, N_a_ and Ĥ, is congruent with the general assumption that larger populations (here FI populations N, S, K, V and CZ population Z) tend to have bigger pool of genotypes/alleles. In large populations, the majority of the individuals were genetically unique, whereas small populations showed higher ratio between N/N_g_. The reduced variation in smaller populations may result from processes such as bottleneck, genetic drift or inbreeding [48, 49]. Nevertheless, several bryophyte studies found no relation between population size and genetic variation [15, 19, 50]. Moderate levels of genetic diversity found in small CZ populations of *C*. *hellerianus* support the earlier views that even small populations are important sources of genetic variation in bryophytes [19, 50] and that such populations may not be drastically threatened by processes causing loss of genetic variation (genetic risk [51]). Interestingly, genetic diversity of the small CZ populations Y and P is somewhat higher than those found in other small CZ populations. Possible explanation could include the history, in course of which these populations experienced significant reduction of population size as a consequence of a severe drop in the availability of substrate. It is known that the tree species composition in the Žofín forest (population P) changed significantly from *Abies alba* dominated forest towards broad-leaved forest dominated by *Fagus sylvatica* with only a minor percentage of spruce (*Picea abies* 15% [52]). The population Y could have benefited from the past or recent gene flow from nearby large population Z, as evidenced by the lowest detected genetic differentiation based on pairwise R_ST_ values (S1 Table).

Fully identical individuals were only found within populations. A large diversity of multilocus genotypes within populations appears to be common in both liverworts [19, 20, 23] and mosses [24, 53], irrespective of the prevailing reproductive mode. The unexpected genetic variation found in taxa with rare sexual reproduction or even in asexually reproducing populations [19–21] implies other sources of genetic diversity than recombination events. The authors mostly suggest neutral somatic mutations, originating in various vegetative parts as the probably most important source. According to Weismann’s doctrine [54], only the germ line (i.e. cells giving rise to gametes) has evolutionary significance and somatic variation within individuals is not transmitted to progeny [55]. However, this is not the true for majority of land plants including bryophytes, as the sequestration of somatic cells and germ line is incomplete, and the extent to which cells or tissues become irreversibly excluded from propagation is rather low [55]. Both sexual organs and asexual propagules are formed in later ontogenetic phases from somatic stem cells, leading to transmission of mutations originated in somatic tissues directly to gametes and/or asexual gemmae or vegetative fragments. In other words, the nature and relative contribution to novel alleles is basically indistinguishable for both sexual and asexual propagules. The propagation of somatic mutations is further enhanced by consistently greater mutation rates in somatic tissues than in germ lines [56]. In plants, as well as in other clonal or modular organisms, such as aphids, freshwater snails, bryozoans, or reef corals, the somatic cells in bryophytes undergo high number of cell divisions before gametes and/or asexual propagules are formed, providing relatively high probability of mutation during numerous DNA replications [57]. In liverworts, a single apical cell is responsible for the shoot growth, and each somatic mutation in this cell is propagated to all thallus parts, which originated from mitotic divisions following the mutation event. Similarly, any somatic mutation that occurred in leaf cells that gave rise to the asexual propagules (gemmae) of liverworts, which often are only 1–2 celled, can easily be directly expressed in the progeny.

Other explanations of remarkable genetic diversity in predominantly and/or seemingly asexual bryophytes may involve e.g. population establishment by multiple genotypes, or periodical occurrence of sexual reproduction generating novel recombinant genotypes. Recruitment of new genotypes from neighboring populations seems to be a rather improbable and rare event in the studied system, as no identical MLG were shared among populations, not even between the spatially closest populations Z and Y, distant only 4 km. Occasional and unobserved sexual reproduction, which might be a major source of variation in large populations with stable reproductive system even with only small number of reproducing individuals per generation [58], also probably plays a minor role in generating the genetic diversity of *Crossocalyx hellerianus*, as the frequency of these events is massively outweighed by the gemmae production. The study [59] reported only 32% of bisexual colonies, and only 12% of colonies producing sporophytes and even these numbers are much higher than in studied Central European populations (only two out of six populations producing perianths at all and 8% of perianth-forming patches in these populations; Table 4). Moreover, the estimated gemmae output per square centimeter of *C*. *hellerianus* colony exceeded the spore production nearly five times, while the ability to germinate in both types of propagules was similar [59]. Prevailing asexual reproduction and absence of recombination in small CZ populations of *C*. *hellerianus* is also indicated by high values of linkage disequilibrium (or P_sex_ values). Significant and rather high linkage disequilibrium was also found in the large FI population N, although the percentage of patches with perianths (25.9%) was comparable with other large FI populations. Nevertheless, the slightly lower genetic variation as inferred from N_g_, N_a_ and Ĥ values was congruent with linkage disequilibrium. This pattern could be explained by low portion of gametophytes arising from sexually produced spores or as a result of inbreeding. Mating may occur among haploid siblings originating from the same sporophyte as a result of non-existing mechanism to distinguish among differently related gametes [60]. Inbreeding would further reduce the relative contribution of otherwise rare sexual reproduction for genetic variation in *C*. *hellerianus*. Especially small CZ populations showed high values of linkage disequilibrium, rather low number of genotypes and aggregation of similar genotypes, which is consistent with the assumption of low recombination efficiency. Therefore, genetic variation in small CZ populations has most likely been caused by somatic mutations, past genetic variation prior to population reduction, and/or establishment by multiple genotypes, although we cannot rule out the contribution of sexual reproduction with respect to the facts discussed above.

Estimates of genetic differentiation among populations reflect the amount of gene flow between them [61]. Isolation by distance inferred from pairwise R_ST_ values was found in most of the studied populations. Genetic differentiation was rather low among the FI populations (R_ST_ values usually < 0.1), whereas the values among the CZ populations mostly exceeded 0.1 (Fig 4). This implies greater gene flow among Finnish populations than it is the case in the Czech Republic, which might be explained by the less fragmented landscape of forests with better availability of decaying wood substrate in Finland. Lesser extent of gene flow among CZ populations can be demonstrated in comparison of genetic differentiation between similarly distant FI and CZ populations. The small CZ population G was considerably differentiated from the 18 km distant Y and Z populations, which is in contrast with low R_ST_ values among FI populations V, S and K, respectively, separated by similar spatial distances (7–18 km). We suppose that suitable substrate, enabling step-by-step dispersal [14, 62] supports gene flow among FI populations in contrast to the complete lack of ‘substrate bridges’ among the CZ populations. Our results are in accordance with other studies of genetic differentiation in wood living fungi and beetles [17, 18]. Generally, habitat loss and fragmentation have negative effect on the genetic structure of populations with respect to the restricted level of gene flow. The combination of reduced gene flow among isolated populations and their reduced size leads to genetic drift and the fixation of different alleles, which brings strong genetic differentiation among populations [48, 49].

### Spatial genetic structure

Direct observations of propagule dispersal in *Crossocalyx hellerianum* [7] showed that a proportion of propagules deposit within few meters from source colonies but a considerable proportion may disperse over farther distances. In the absence of any specialized dispersal adaptations, the wind probably serves as the main dispersal vector, and the deposition of propagules may be further enhanced by water during rainy days. Dispersal by animal vectors such as the ants, hardly has an important role [23]. Anyway, the direct methods have limited use for large spatial scale studies (few hundreds of meters) or for studies on short timescale. In these cases, indirect methods revealing the spatial genetic structure can bring a reasonable assessment of propagule dispersal.

High values of kinship coefficients observed in most of the small populations provided the evidence for aggregation of similar genotypes. This can be caused by the relatively low level of genetic diversity resulting from bottleneck and/or founder effect, prevailing asexual reproduction or breeding of related individuals, as discussed above. Spatial distribution reflects both substrate availability and the mode of reproduction. If suitable habitats are evenly distributed and spore production is frequent, allowing effective dispersal at the middle and long distances, randomness in distribution, reflected in the absence of SGS, can be achieved [33]. This is not the case at localities with small populations of *C*. *hellerianus*, where the amount of decaying wood is generally low and essentially all available substrate is occupied. Random SGS cannot be achieved in the absence of sexual reproduction, evidenced by high values of linkage disequilibrium and absence of perianths in small CZ populations. Asexual reproduction by gemmae represents here the most important and efficient role in maintaining the populations. This is in agreement with previously postulated conclusions in vascular plant studies [63, 64].

Recent investigations of SGS in seed plants, reviewed in [65] showed that its presence is positively correlated with self-compatibility, low population densities, and poorly dispersed seeds. In *C*. *hellerianus*, large populations possessed less SGS than small populations, with their individuals showing marked decrease in kinship over 50 cm distances and appearing to be without any obvious kinship on distances exceeding 16 m. This result reflects higher population density and more frequent spore production observed in large populations, both allowing more efficient dispersal of different or novel MLGs on farther distances, which reduces the pattern of SGS. Anyway, even in large populations, the plants continue to produce gemmae massively, contributing to aggregation of genotypes and presence of SGS over short distances. Vegetative reproduction by gemmae obviously contributes to economic balance avoiding the costly production of sporophytes [59].

Comparison of SGS shape between studied populations of *C*. *hellerianus* and the small-scale pattern of SGS in a closely related liverwort species, *Barbilophozia attenuata* [23] shows similar patterns between large CZ and FI populations and the shape for *B*. *attenuata*, whereas small CZ populations of *C*. *hellerianus* differed in noticeably strong SGS. Whereas the kinship coefficients reached zero over 8–10 m in *B*. *attenuata*, they approached zero not earlier that at distance of 16 m and turned negative at distance of 500 m in *C*. *hellerianus*, reflecting the aggregation of genotypes over larger distances in the latter species. This might infer that *B*. *attenuata* produces sporophytes more often or the gemmae of *C*. *hellerianus* have better dispersal capacity. The latter explanation can be supported by the difference in propagule weight, because the smaller gemmae of *C*. *hellerianus* have about eight times smaller volume than the gemmae of *B*. *attenuata*.

In our study, clones, probably arising from gemmae, were detected even at distances of 20, 50, 62, and 80 m. Although some of the identical MLG may have arisen from sexual reproduction, the probability of such events was negligible in large FI and CZ populations (S4 Fig). Higher frequency of clones distributed over long distances in FI populations thus probably reflects the larger spatial extent of these populations. The observation of clones spanning long distances is consistent with the results of an earlier experiment [7], who found considerable potential for long-distance dispersal of gemmae in *C*. *hellerianus*. We observed most of clones to be dispersed only within logs at short distances in large populations, whereas small CZ populations showed significant portion of clones dispersed at distances up to 10 m (Fig 6). The apparently more efficient dispersal of clones in small CZ populations might however rather be the consequence of the absent sporophyte production. On the other hand, the clonal pattern of FI populations seemingly involving long distance dispersal might be a consequence of several successive step-by-step dispersal events over much shorter distances, as the continuous availability of epixylic substratum in space and time at Finnish localities increases the probability of successful establishment.

## Conclusions

Genetic diversity in populations of the dioicous epixylic liverwort *Crossocalyx hellerianus* was related to population size but even the small populations were found to be important sources of genetic variation. Recombination connected with sexual reproduction only plays a significant role in generating the genetic diversity in large populations of *C*. *hellerianus*, whereas smaller populations are maintained by vegetative diaspores and their main source of genetic diversity are probably the somatic mutations. We were able to demonstrate notably low levels of gene flow among populations in Central Europe, where habitat fragmentation poses a significant barrier to dispersal of diaspores. Populations from southern Finland show lower levels of inter-population differentiation at the same distances, which can probably be explained by the presence of step-by-step dispersal. The fine scale study of SGS revealed a strong aggregation of genotypes, particularly in smaller populations, and at the same time showed that asexual reproduction is an efficient mean of maintaining the population at not only the short distances, given the spatial extent of clones spanning dozens of meters. On the other hand, strong SGS in large populations seems to be reduced by the relatively efficient dispersal of both spores and gemmae.

## Supporting Information

S1 Fig Sampling sites in the Czech Republic and Finland. Abbreviations of localities correspond to Table 1. Made with Natural Earth. Free vector and raster map data @ naturalearthdata.com.
(TIF)

S2 Fig Multilocus genotypic resolution of microsatellites in the data set of *Crossocalyx hellerianus*. The plot was generated using 1,000 random samples of 1 to 9 loci. Resampling of loci indicated that our set of nine loci had sufficient haplotypic resolution, as even the use of approximately 7 loci would reveal the majority of MLGs detected in this study.
(TIF)

S3 Fig Number of distinct multilocus genotypes (MLGs) plotted against the number of individuals (A and B). Plots were generated for each population separately (A) small populations and (B) large populations, using 1,000 random samples of individuals to see if the relationship reached a plateau. Resampling of individuals indicated that increased sampling would yield higher number of MLGs in large populations (B), whereas in small populations the number of MLGs mostly tended to reach a plateau (A). The estimated number of MLGs was substantially lower in small populations (1–8 MLGs) than in large populations (5–15 MLGs, grey part of B) at smaller sampling sizes (N ranging from 5 to 16), corresponding to the maximum sampling size in small populations. Therefore, sampling in small populations was probably rather comprehensive despite lower number of individuals in population, whereas in large populations the clonal diversity estimates could be underestimated.
(TIF)

S4 Fig The probability of sexual reproductive events. Probability of sexual reproduction (P_sex_) was plotted against the particular repeated multilocus genotypes (MLG) for populations (A) small CZ populations, (B) large CZ population and (C) large FI populations. If the probability is below significance threshold (P_sex_ < 0.05), the respective individual is not likely to be the result of a distinct event of sexual reproduction. Thus we can conclude that individuals with identical genotypes, which occur more than once in the population and their P_sex_ < 0.05, were probably established from asexual propagules (predominantly found in large CZ and FI populations—Z, N, S, K and V).
(TIF)

S1 File Certification of Ethics statement. (DOCX)

S2 File Allelic data for all samples. Abbreviations of localities correspond to Table 1. Samples within each population were numbered, and individuals collected within a single patch were indexed by letters A-F. Missing data were assigned as ‘-1’.
(XLSX)

S3 File Distances among sampled individuals within each population. Abbreviations of localities correspond to Table 1. Samples within each population were numbered, and individuals collected within a single patch were indexed by letters A-F.
(XLSX)

S1 Table The pairwise R_ST_ values calculated between all populations. Significance of *F* values is marked as *** *P* < 0.001; ** *P* < 0.01; * *P* < 0.05.
(DOCX)
