# Supplementary figures and images for: Comparison of Genetic Structure of Epixylic Liverwort Crossocalyx hellerianus between Central European and Fennoscandian Populations
Source: PLoS One. 2015 Jul 17;10(7):e0133134. doi: 10.1371/journal.pone.0133134 (PMC4505853; doi:10.1371/journal.pone.0133134)

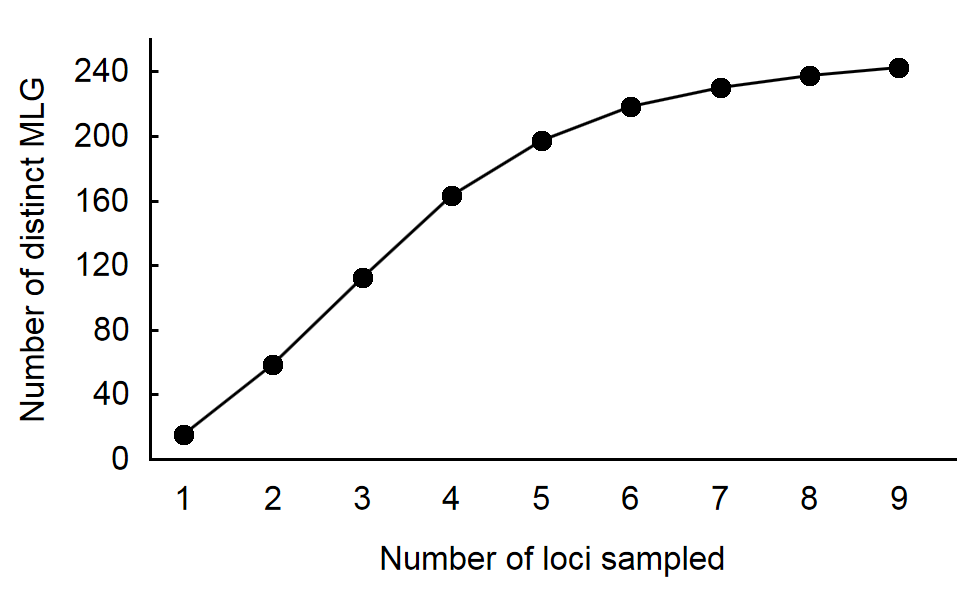

Supplement: S2 Fig — The plot was generated using 1,000 random samples of 1 to 9 loci. Resampling of loci indicated that our set of nine loci had sufficient haplotypic resolution, as even the use of approximately 7 loci would reveal the majority of MLGs detected in this study. (TIF) [file pone.0133134.s002.tif]

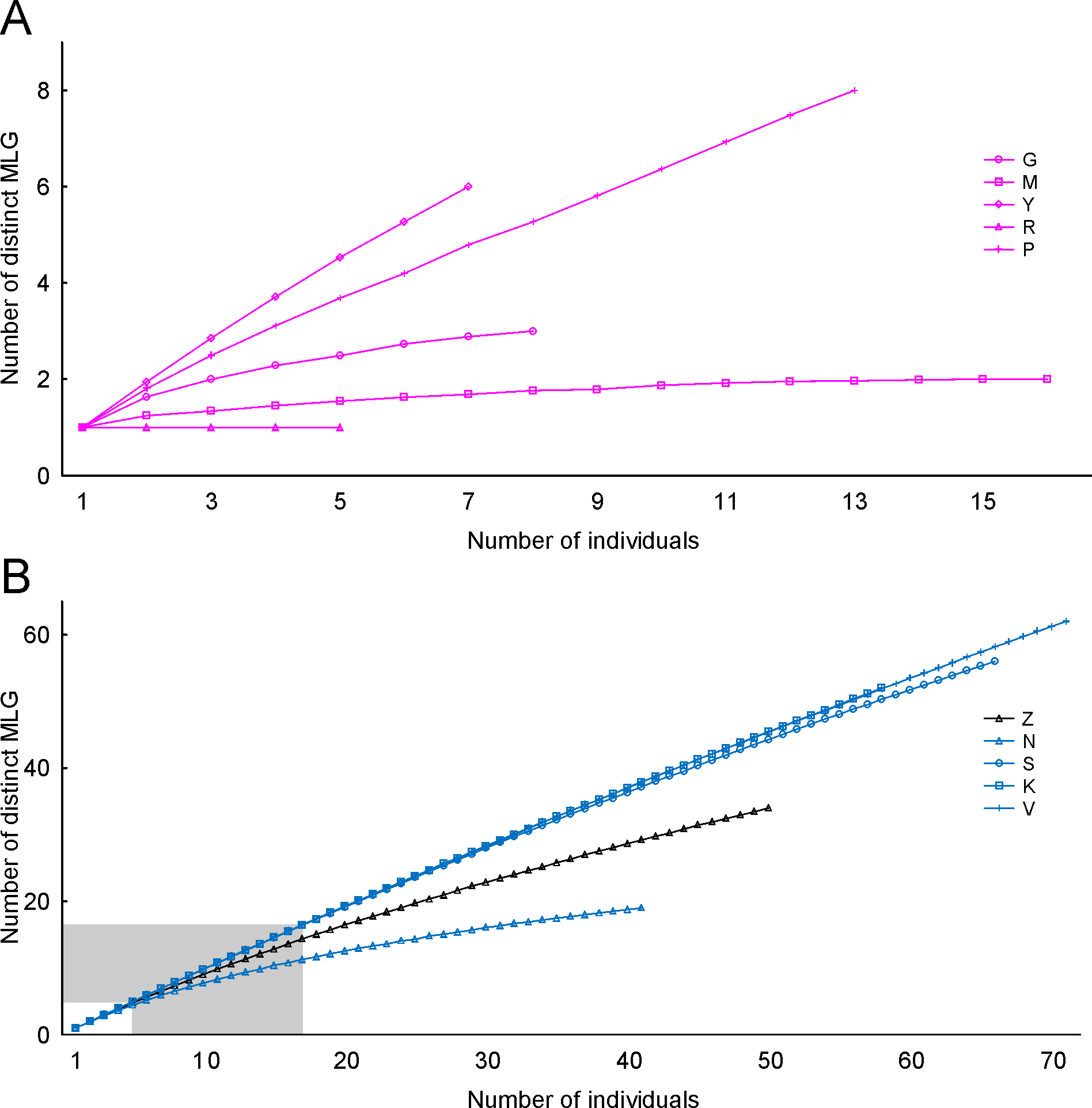

Supplement: S3 Fig — Plots were generated for each population separately (A) small populations and (B) large populations, using 1,000 random samples of individuals to see if the relationship reached a plateau. Resampling of individuals indicated that increased sampling would yield higher number of MLGs in large populations (B), whereas in small populations the number of MLGs mostly tended to reach a plateau (A). The estimated number of MLGs was substantially lower in small populations (1–8 MLGs) than in large populations (5–15 MLGs, grey part of B) at smaller sampling sizes (N ranging from 5 to 16), corresponding to the maximum sampling size in small populations. Therefore, sampling in small populations was probably rather comprehensive despite lower number of individuals in population, whereas in large populations the clonal diversity estimates could be underestimated. (TIF) [file pone.0133134.s003.tif]

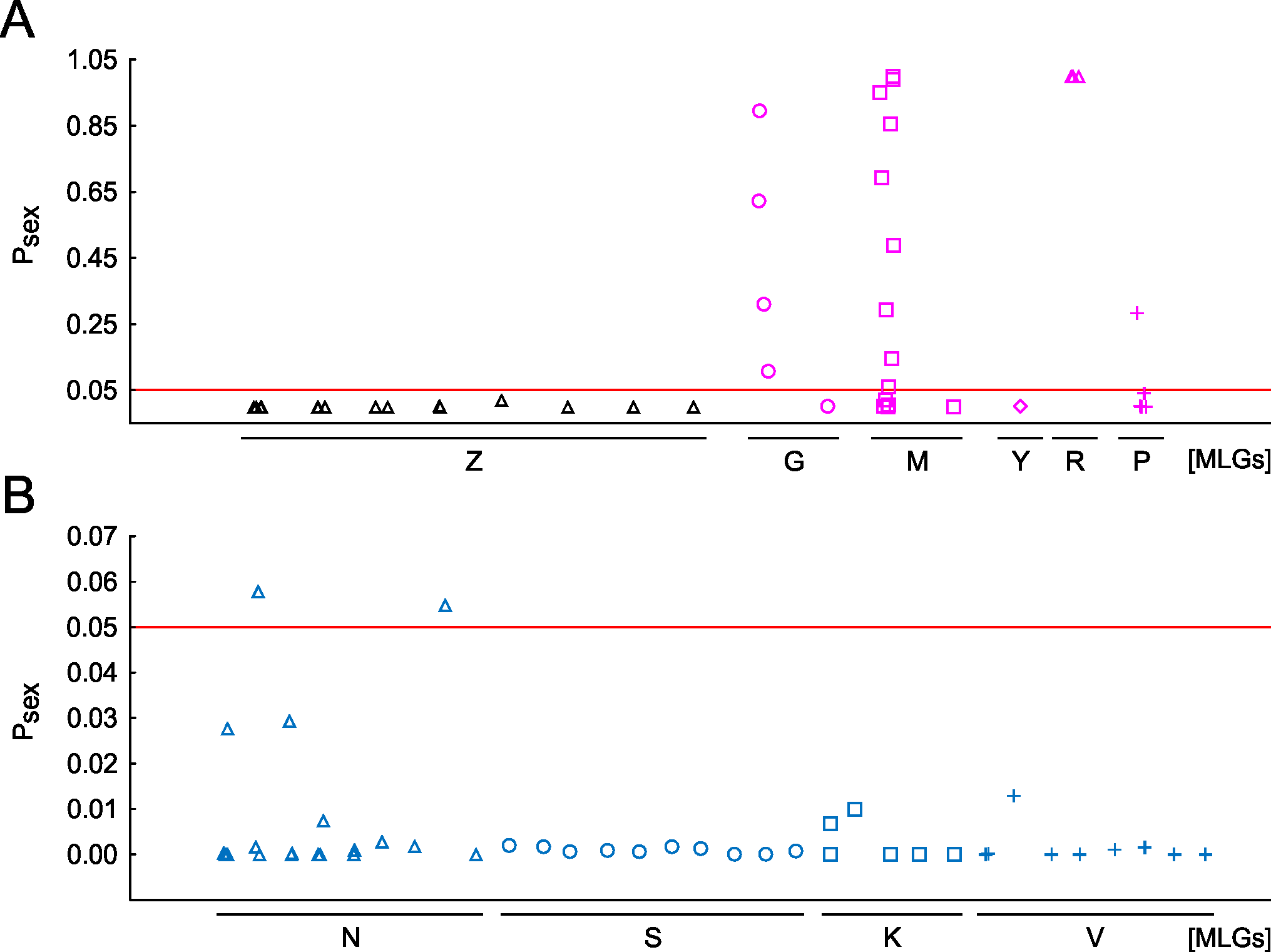

Supplement: S4 Fig — Probability of sexual reproduction (Psex) was plotted against the particular repeated multilocus genotypes (MLG) for populations (A) small CZ populations, (B) large CZ population and (C) large FI populations. If the probability is below significance threshold (Psex < 0.05), the respective individual is not likely to be the result of a distinct event of sexual reproduction. Thus we can conclude that individuals with identical genotypes, which occur more than once in the population and their Psex < 0.05, were probably established from asexual propagules (predominantly found in large CZ and FI populations—Z, N, S, K and V). (TIF) [file pone.0133134.s004.tif]

**S1 File.** **Certification of Ethics statement.**


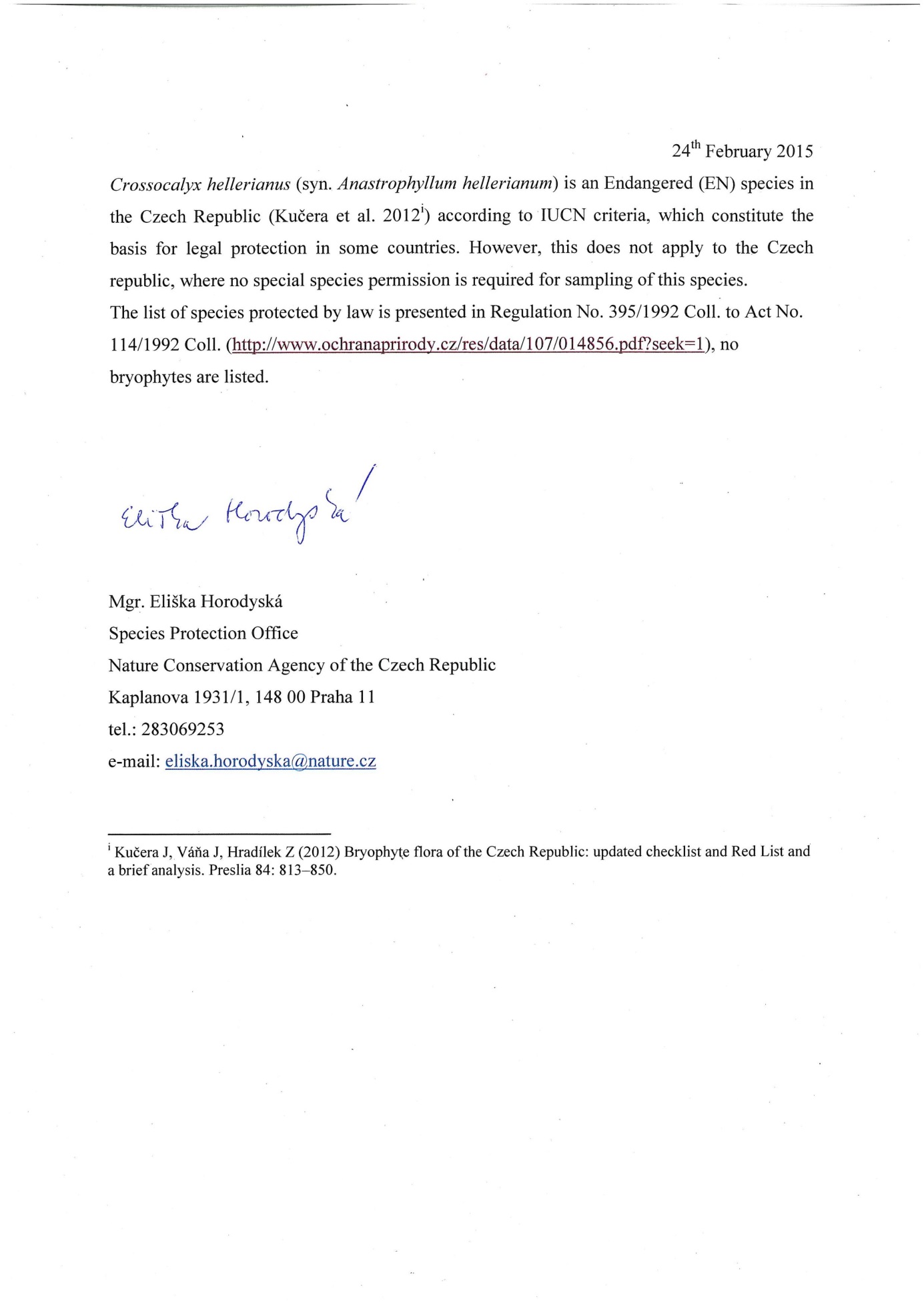

Supplement: S1 File — (DOCX) [file pone.0133134.s005.docx]
