# Supplementary material for: Comparison of Genetic Structure of Epixylic Liverwort Crossocalyx hellerianus between Central European and Fennoscandian Populations
Source: PLoS One. 2015 Jul 17;10(7):e0133134. doi: 10.1371/journal.pone.0133134 (PMC4505853; doi:10.1371/journal.pone.0133134)
Supplement: S1 Table — Significance of F values is marked as *** P < 0.001; ** P < 0.01; * P < 0.05. (DOCX) [file pone.0133134.s008.docx]

**S1 Table.** The pairwise R_ST_ values calculated between all populations.

| **Locality** | **Z** | **G** | **M** | **Y** | **R** | **P** | **N** | **S** | **K** |
| --- | --- | --- | --- | --- | --- | --- | --- | --- | --- |
| **G** | 0.172*** |  |  |  |  |  |  |  |  |
| **M** | 0.179*** | 0.415*** |  |  |  |  |  |  |  |
| **Y** | 0.003 | 0.129** | 0.176** |  |  |  |  |  |  |
| **R** | 0.432*** | 0.777*** | 0.656*** | 0.192 |  |  |  |  |  |
| **P** | 0.076* | 0.153** | 0.256*** | 0.051 | 0.740*** |  |  |  |  |
| **N** | 0.396*** | 0.575*** | 0.501*** | 0.279*** | 0.152* | 0.515*** |  |  |  |
| **S** | 0.328*** | 0.515*** | 0.487*** | 0.235*** | 0.323** | 0.464*** | 0.169*** |  |  |
| **K** | 0.208*** | 0.356*** | 0.367*** | 0.083* | 0.161* | 0.317*** | 0.139*** | 0.053** |  |
| **V** | 0.186*** | 0.333*** | 0.325*** | 0.080* | 0.143* | 0.281*** | 0.083** | 0.037* | -0.001 |

Significance of *F* values is marked as *** *P* < 0.001; ** *P* < 0.01; * *P* < 0.05.
